# Proceedings of the Iowa State Dental Society

**Published:** 1878-10

**Authors:** 


### Proceedings of Societies.

### PROCEEDINGS OF THE IOWA STATE DENTAL SOCIETY.

The sixteenth annual meeting of the Iowa State Dental Society was held in Iowa City, Tuesday, July 17, 1878. The sessions were held in the medical college rooms of the university. At the opening twenty-four members were present.

The Society was called to order by Dr. R. D. Cochran, Vice-president. An address of welcome was delivered by Dr. Clapp, M. D., of Iowa City. The Doctor, in his remarks, congratulated the dental profession in attainments and rapid progress made hitherto, and that the days of quackery and incompetency were rapidly passing away. The profession of dentistry, he said, is now regarded as nearly on a level with the profession of medicine. The people of this city are gratified that this meeting is to be held here. We will be glad to have you bear with you pleasant remembrances of your visit here.

Dr. J. T. Abbott made a very happy response to the address of welcome.

After some congratulatory remarks by Dr. Ingersoll, of Keokuk, the Society adjourned till two o'clock.

### FIRST DAY—AFTERNOON SESSION.

The Society met according to adjournment, with an increase in the number present. The treasurer presented his report as follows:

| | |
|---|---|
| Receipts during the year | $207 86 |
| Disbursements | 62 50 |
| Amount on hand | 145 36 |

The President, Dr. G. S. Shattuck, formerly of Belle Plain, now of Detroit, Mich., not being present his address was read by the Vice-president, R. L. Cochran, of Burlington. (*This address is published on another page of this number of the* REGISTER.—EDITOR).

Dr. Dickinson, of Dubuque, and Dr. Mullett, of Clinton, both presented their methods of registering dental operations.

Dr. J. Hardman, of Muscatine, then read a paper setting forth his views of the use of Arsenious acid in dentistry, after which the subject was fully discussed.

On recommendation of the committee on membership Dr. George Gibson, of Eldora, was elected to active membership and Dr. E. S. Taylor, of Wyoming, and Dr. N. L. McConn, of Decotah, to junior membership.

Dr. Ingersoll, of Keokuk. in speaking of sensitive dentine said that in his hand, moistening the sensitive surface with Creasote and then thoroughly burnished, had proved efficient.

The professors of the university and the medical profession of the city were invited to attend the meetings of the Society.

Adjourned till eight o'clock, p. m.

EVENING SESSION.

The Society met according to appointment. The selection of the next place of meeting was now in order. After some discussion it was decided to hold the next annual meeting at Clear Lake,

Adjourned.

WEDNESDAY MORNING.

The entire forenoon session was devoted to clinics and microscopic observations.

The clinics were conducted by Dr. Fuller, of Des Moines; Dr. Denise, of Burlington; Dr. L. C. Ingersoll, of Keokuk, and Dr. Dickenson, of Dubuque.

Adjourned.

WEDNESDAY AFTERNOON SESSION.

The Society met at two o'clock. The first order of business was the election of officers, by which the following were chosen: President, Dr. R. L. Cochrane, of Burlington; Vice-president, Dr. I. D. Pearce, of Iowa City; Corresponding Secretary, Dr. L. C. Ingersoll, of Keokuk; Recording Secretary and Treasurer, Dr. E. E. Hughes, of Newton.

A paper was now read by Dr. L. C. Ingersoll. Subject: "Diagnosis." (*For which see another page of this number.*—EDITOR).

This paper called out a genial expression of endorsement from those present.

Dr. Tulloss said he thought the subject had been well and exhaustively treated, and all that was necessary had been said.

Dr. Wilson, of Burlington said the paper was very interesting and had opened a new field of thought for him, He had not been in the habit of working in the manner pointed out—had not always made a proper diagnosis of the case before him. He regarded the paper a very valuable one.

Dr. Kulp stated that he for many years, to some extent had practiced the plan of diagnosis presented. He did not consider the pulp of a tooth necessary, and in sixteen years practice in one locality his experience demonstrates the truth of the proposition. In cases of abrasion, the pulp may be wholly abstracted without pain. Ten years hence the treatment of pulp will be different from that of the present. Some have said, "Dr. Ingersoll is stumbling upon the truth."

Dr. Chase, of St. Louis, endorsed the paper by Dr. Ingersoll, and thought it was of great interest as it aroused opposition from high sources. Dr. Taft has stated there were many who would take issue with the Doctor and claim that the nerve of a tooth is obsolutely necessary. The question has two sides and should be discussed at length and considered by each one for himself.

Dr. Mullett said that Dr. Atkinson had stated: "Who destroyed the nerve or pulp was weak or wicked," and asked if any one knew that he was of the same opinion still.

Dr. Ingersoll stated that Dr. Atkinson said "it was a crime to destroy the nerve."

Dr. Abbott thought "Dr. Atkinson was on the anxious seat."

Dr. Kulp was of the opinion that where the pulp was perfectly obliterated, as nature does it, and then properly filled there would be no trouble with the filling.

Dr. Tulloss asked if it was possible always to make a filling so there would be no cavity.

On motion discussion upon the subject presented was deferred till aftei reading of other essays on subjects nearly related to the one first presented.

Dr. I. P. Wilson, of Burlington, read the next paper on "Material for Filling Teeth," after which Dr. I. D. Pearce' of Iowa City, presented an able paper upon "Why do our Fillings Fail." After the reading of this paper, it was agreed that Dr. Chase, of St. Louis, have the earliest opportunity to present his views upon the recent experiments made by him. Thursday morning was set apart for that purpose.

On motion the Society adjourned till eight o'clock to-morrow morning.

THURSDAY MORNING SESSION.

The Society met according to adjournment. An hour was now devoted to visiting the various departments of the university. At half-past nine the Society resumed its regular business.

Dr. Hardman was now called upon and addressed the Society for a short time; he alluded in a very complimentary way to the papers that were read on yesterday, congratulating the members on the evidences of progress that seem so abundant and so full; he suggested by these more encouraged and stimulated to press forward. At the close of these remarks Dr. Chase took the floor and presented his views on plastic fillings, embodying about the same as he has elsewhere set forth. At the conclusion of Dr. Chase's remarks, Dr. Kulp asked: If we accept the proposition laid down what shall we do? Dr. Chase replied: No one is satisfied; we want some plastic filling that is not harder than the teeth and will not be acted upon, just the same as the enamel of the teeth.

Dr. Ingersoll, of Keokuk, being called upon said: I shall have to confess my ignorance upon this subject. My knowledge of chemistry dates back to twenty-five years ago, and not having much time from my regular professional duties I have left these investigations to others. We must all acknowledge that there are certain inherent principles in nature over which we have no control, and when an honest man places himself in communication with nature and he believes she has revealed certain things to him we should accept them as true until time has proved them false. It should be remembered we look at things from different stand points and in different lights. Such men as Prof. Tyndall, from certain experiments, deduced certain principles which for some time were supposed to be true, but afterwards the mistake was discovered and confessed by that eminent scientist. We should be very careful in our experiments and not be too easily convinced. I am of the opinion that the experiments made have not been sufficient to convince, and I am not yet willing to accept the propositions. I am open to conviction. My experience in my profession does not correspond with the experiments of Dr. Chase. I say gold is far superior to amalgam in most cases, but have seen cases where I thought amalgam better than gold. In my practice I remove more amalgam fillings than gold How this may be explained I know not. That there is galvanic action in the mouth I do not doubt,

Dr. Pearce, of Iowa City, remarked: I have the egotism to criticise Dr. Chase's experiments a little—There is a law of galvanic action. If you ignore electrical action you ignore the teachings of all experiments. By diagrams on the blackboard he explained how if two magnets, both positive or both negative they would be attracted one to the other. And he did not consider Dr. Chase's experiment a proper test because of the complex galvanic action produced by all the cubes with fillings being placed in the same solution in one vessel. If electricity is the basis and chemical action is caused by electrical action we must accept Dr. Chase's theory.

In my experiments I used principally the enamel of the tooth (because that is the only part exposed in the mouth) and prepared polished cubes to which I attached with a silk cord polished plugs of gold and amalgam. These cubes I soaked for a week in water, weighed carefully to a quarter c. g. and with the plugs ligatured to the cubes I suspended in separate bottles one-third full of acetic acid. In this condittion they remained one week, after which they were removed and carefully weighed. It was found in some cases, several experiments being made, that the cube with gold attached lost more than the cube to which the amalgam was attached, but in no instance was the loss found in the proportion reported by Dr. Chase. In most cases the loss of each was found to be about the same. Prof. Faraday demonstrated the principle that it was not necessary for galvanic action to have the substances in contact, and as in Chase's experiment the cubes were all in one solution or in contact, is not my experiment a better test than his? Dr. Chase claims "as the teeth need saving the most gold is the worst filling," In my judgment this does not belong to the so-called "New Departure." The doctor himself practiced this thrity years ago. Since coming to Iowa City I have found gold fillings, the work of Dr. Chase fully thirty years ago, and they will likely last ten or fifteen years longer. This proves that Dr. Chase was a good gold filler years ago and if he should, with his present experience and skill according to the improvements of the profession, put in gold fillings, is it not reasonable to suppose they would last for a hundred years?

Dr. Chase asked: Why do gold fillings last so long? I believe it is because of thorough work and healthy condition of the mouth. Pure putty would have done just as good service if not injured by the wear of mastication. The time of lasting depends upon the condition of the mouth.

Dr. Pearce asked if decomposition of the teeth was due to animal electricity.

Dr. Chase replied that it was not. Fillings fail because of inperfect work, leaks, and the favorable condition for galvanic and chemical action. Electrical action may sometimes be the primary cause.

Dr. Pearce said: One more question; I am after the truth; I advocate thoroughness; I do not believe gold should be used in all cases and we can not get along without amalgam. If we are thorough and the gold filling is perfectly made why will it not be permanent?

Dr. Chase replied: That is just where the trouble lies. None of us do perfect work. It is impossible. My exper ment is not a belief, it is a knowledge. No man is convinced of a thing until he is forced to. I don't care tor my reputation, I do not care for consistency. I do not care what I believed years ago. It is absurd to keep in the old ruts simply to be consistent. I do not force these conclusions upon any one. I simpy give them for what they are worth. They will set you all to thinking, and you will study the subject, and in this way we will get at the truth. I do not know everything, but if I can answer any question that may be propounded I will be pleased to do so.

Dr. Crawford asked: What is the cause of decay in teeth?

There are too many causes to now enumerate, but the acid condition of the saliva is the principal cause.

Dr. Wilson suggested that there was a misunderstanding as to the meaning of Dr. Chase's proposition. "As teeth need saving most, gold is the worst filling."

Dr Tulloss said: I am glad Dr. Chase came to us this year. We in our conventions had gone over the whole ground and in our practice had become somewhat stereotyped. I was prejudiced against this new departure, but I am willing to learn.

I too for twenty-five years have wanted a new filling something plastic, something the color and hardness of the teeth. I believe in thorough work, and that a good amalgam filling is better than a poor gold one. In some cases owing to the peculiar location and condition of the tooth we can not use gold successfully and amalgam is best. We must continue our experiment and diagnose our cases thoroughly. We must go on and make progress in our profession. We must not ride a hobby, and must always use good judgment. When I first began to practice I was taught it was a sin to use amalgam. Now in one-half of the teeth filled amalgam is used.

Dr. Ingersoll asked if there was any insurmountable difficulty to making the experiments in the mouth so to know what effect, if any, vitality would have in the case.

Dr. Chase thought it impossible to make an accurate experiment in the mouth. He thought they had thousands oexperiments approximating the truth in the mouths of all who had teeth filled with metals.

Dr. Hardman asked if experiments had ever been made with gold alone.

Dr. Chase thought possibly that he had, but did not remember, if he had he did not consider them reliable.

Dr. Ingersoll stated that he alluded to the influence of vitality because we are familiar with its influence upon seeds taken into the stomach, and also in woody substances. Possibly the electrical condition of the mouth would neutralize galvanic action produced in the experiment as given. It was once thought very wrong to put gold and amalgam in the same tooth. I have seen gold and amalgam filling in contact in the same tooth do good service and not in a healthy mouth either.

Dr. Pearce again went to the black board and made a more detailed report of his experiments and showed in what particular they were superior to those made by Dr. Chase. In the experiments of the latter the cubes with their respective fillings were all placed in the same solution and only half an inch apart, consequently 'gold being positive as to all the other metals in the solution, and the solution being a conductor, a complex galvanic action was produced and the action or loss of the cube containing the gold plug was much greater than it would have been if placed under the circumstances adopted in the experiments made by Dr. Pearce. He prepared the acid solution in a reservoir, from which at the same time he prepared his smaller bottles for experiments; each cube and plug was weighed, prepared and experimented upon at the same time in different bottles and under the same conditions.

Dr. Kulp remarked that he endorsed the words of Dr. Ingersoll and was of the opinion that neither of the experiments proved anything for any filling. In the mouth I think a different condition of things would be found than in the manner the experiments were made. I think the difficulty with our fillings is in our manipulating If we could all remain young, vigorous and ambitious we would have better success,

Amalgam is so much easier manipulated than gold is the reason it is thought to be a superior filling. My twenty years' experience does not corroborate the experiments of Dr. Chase. I can not believe amalgam is better than gold. I am willing to accept new principles when adduced and proven and to accept all that is good in these experiments, and if it is true that acid or galvanic action in the mouth is the trouble then let us try and find some remedy. Let us get a dentrifice that will prevent action on the teeth. For twenty years I have used a soap that seems to answer the purpose of cleansing the teeth, and possibly a good dentifrice is all that is necessary. The only thing I censure Dr. Chase for, is the manner in which he has put these new ideas before the world. I am personally a warm friend of the doctor and have been for years, but by the issue raised I think he fails to appreciate the back bone he has given to quackery. This new departure has a tendency to tear down the profession and to build on its ruius a system of empiricism. I do not think Dr. Chase should make such extravagant statements. I have no objections to experimenting if everything is all right. If quackery should come in and take away our business we would feel life had been a failure. I am in favor of being directed to a truth if it is a truth that will be of benefit, but feel as though such things should be received cautiously, and such statements should be made in private before giving them to the world.

Dr. Andrews said: With due deference to Dr. Kulp, I believe the statements of Dr. Chase were made in good faith and with a view of doing us good. It will awaken a spirit of inquiry in the profession. I believe in progress and believe ten years hence will develop great improvements. I do not say a word against the old school, but will confess I am pleased with the presentation of the subject before us.

Dr. North said: I have great interest in the presentation and discussion of the subject. I, at one time, considered a man who would use amalgam unworthy a place in the profession; but now I do not believe we could practice dentistry successfully without the use of amalgam.

Dr. Hallet asked: If animal electricity is present in the mouth and decay follows as the result, how can we ever expect to have permanent fillings? We should then get a preventive for this decay. I believe in giving the truth free course; error will find its place and die a natural death.

Dr. Tulloss said: Though I do not endorse all that has been said I believe in saying what one honestly believes to be the truth. I am an advocate of gold, but not an extremist.

AFTERNOON SESSION.

At two o'clock the Society met pursuant to adjournment. President Pickard being invited to make a few renarks, he said: I came in because I like to be with men who are striving to advance. I like those men who are dissatisfied with present attainments. I assure you that your Society will always be welcome to the use of our rooms, our cabinet and our lectures. We hope that sooner or later we may have a dental department connected with the university. I shall use my influence to bring about a state of affair so desirable.

Upon motion the society proceeded to the election of essayist for next year. which resulted as follows: L. C. Ingersoll, E. R. Nullett, W. O. Kulp, S. T. Abbott, E. E. Hughes, I. D, Pearce, J. Hardman, I. P. Wilson.

On motion the Secretary read the names of delinquent members. A motion was made to drop delinquent members from the Society roll, but by amendment Art. 5th of By-Laws was suspended until the next regular meeting of the Society. The Secretary was instructed to notify all delinquent members of the action of the Society.

Upon motion the following persons were appointed to act as legislative committee: L. C. Ingersoll, W. O. Kulp and I. D. Pearce.

Upon motion the following committee was chosen to endeavor to secure a dental department in connection with the University: I. D. Pearce, L. C. Ingersoll, I. P. Wilson, N. H. Tulloss and J. Hardman.

Upon motion L. C. Ingersoll, of Keokuk, was chosen the delegate to the American Dental Association, and $50.00 were appropriated to defray his expenses, but that the appropriation shall not be a precedent.

The question box was opened and the first question discussed was: "Is it admissible to make an amalgam filling by the side of a gold one, especially near or under the gums?" Dr. Chase answered, yes—even though the metals come in contact.

Second question: "What are the pathological changes occurring in the formation of alveolar abscess?" Dr. Ingersoll replied: If the abscess is occasioned by death of the pulp it causes inflammation of the root membrane, then congestion of the capillaries and degenerate blood which is then effused as pus between the cementum and the peridental membrane, the accumulation of pus is gathered in a sack at the apex of the root till by pressure, it induces absorption of the wall of the alveolus and is prevented from distribution into the soft tissues by the formation of a canal and a sinus on the surface of the gums where the pus is generally discharged.

Third question: "Does the premature extraction of deciduous teeth affect the jaw?" Dr. North replied that he did not think it did.

Fourth question. "Does the abscess of a tooth always prove the death of the pulp?" W. O. Kulp answered, Yes!

Fifth question. "How to treat exposed pulp?" Dr. I. P. Wilson replied, If it is but slightly exposed, cap and fill. I generally prefer to fill temporarily.

Sixth question. "What is the best treatment for alveolar abscess?" Carbolic acid was suggested as the best remedy. To be used not with a syringe, but by forcing the medicine into the hollow of the tooth by the use of a small piece of rubber.

Upon motion the Treasurer was instructed to pay the gas bill, and the society adjourned to meet next year at Clear Lake.

